# Characterization of Proanthocyanidins from *Parkia biglobosa* (Jacq.) G. Don. (Fabaceae) by Flow Injection Analysis — Electrospray Ionization Ion Trap Tandem Mass Spectrometry and Liquid Chromatography/Electrospray Ionization Mass Spectrometry

**DOI:** 10.3390/molecules18032803

**Published:** 2013-03-01

**Authors:** Viviane Raïssa Sipowo Tala, Viviane Candida da Silva, Clenilson Martins Rodrigues, Augustin Ephrem Nkengfack, Lourdes Campaner dos Santos, Wagner Vilegas

**Affiliations:** 1 Department of Organic Chemistry, Institute of Chemistry, UNESP—Sao Paulo State University, 14800-900, Araraquara, Sao Paulo, Brazil; 2 Department of Organic Chemistry, Faculty of Science, University of Yaounde I, PO Box 812, Yaounde, Cameroon; 3 Center of Chemical and Instrumental Analysis, Embrapa Agroenergy, PqEB, W3 North, 70770-901, Brasilia, Distrito Federal, Brazil; 4 Experimental Campus of Sao Vicente, UNESP—Sao Paulo State University, 11350-000, Sao Vicente, Sao Paulo, Brazil

**Keywords:** HPLC/ESI-MS, FIA-ESI-IT-MS^n^, proanthocyanidins, *Parkia biglobosa*, Fabaceae, antiradical

## Abstract

The present study investigates the chemical composition of the African plant *Parkia biglobosa* (Fabaceae) roots and barks by Liquid Chromatography - Electrospray Ionization and Direct Injection Tandem Mass Spectrometry analysis. Mass spectral data indicated that B-type oligomers are present, namely procyanidins and prodelphinidins, with their gallate and glucuronide derivatives, some of them in different isomeric forms. The analysis evidenced the presence of up to 40 proanthocyanidins, some of which are reported for the first time. In this study, the antiradical activity of extracts of roots and barks from *Parkia biglobosa* was evaluated using DPPH method and they showed satisfactory activities.

## 1. Introduction

The African Locust Bean (*Parkia biglobosa*) [1], belongs to the Fabaceae family. Its natural distribution extends from Senegal and Guinea in Western Africa eastwards to Uganda [2]. The fermented seed (known as dawadawa or soumbala) is a popular, protein-rich condiment in many Western African countries [3,4]. Studies with species of the genus *Parkia* showed that most of them have antioxidant [5], antimicrobial [6,7], gastroprotective [8] and some antihypertensive effects [9]. Previous phytochemical investigations of *P. biglobosa* reported that sterols, triterpenes [10], catechins and phenolic derivatives could be isolated from the bark [11], and fatty acids from the seed oil of this species [12]. From other species of *Parkia*, iridoid glucosides [13], tannins [14], anthocyanidins [15] and flavonoids [16] were was isolated.

Proanthocyanidins (PAs) are also named condensed tannins, and are known as the second most abundant class of natural phenolic compounds after lignin [17]. They are polyphenol or polyhydroxyflavan-3-ol oligomers and polymers linked by carbon-carbon bonds between flavanol subunits [18]. Condensed tannins are usually linked to each other through C4–C8 or C4–C6 B-type interflavanoid bonds and sometimes, an A-type interflavanoid bonds is observed when an additional ether linkage is formed between C2 and O7, leading to B-type and A-type proanthocyanidins, respectively [19]. B-type proanthocyanidins are sometimes esterified with a gallic acid to form a gallate moiety [20] or with glucosyl substituents [19]. Condensed tannins are subdivided into various groups including: procyanidins, considered as the largest class of proanthocyanidins, which are oligomers and polymers of (*epi*)catechin subunits; prodelphinidins, which are made up of (*epi*)gallocatechin units; and propelargonidins, constituted of (*epi*)afzelechin flavan-3-ol units [21,22]. The biological activities of condensed tannins are generally related to their most important property: the degree of polymerization (DP). The activities increase proportionally with the raise of the DP [23]. Proanthocyanidins is very important in human health protection and disease prevention; they have been reported to be strong antioxidants [24,25], anticancer [26], anti-ulcerogenic [27], antiprotozoal [28], cicatrizing [29], anti-inflammatory [30] agents and other beneficial properties that can suggest the application of species with tannins in pharmacologic studies, like anti-HIV and anti-HSV effects [31] and pancreatic lipase inhibition properties [32].

An improved analytical method based on high-performance liquid chromatography coupled to electrospray negative ionization—ion trap mass spectrometry (HPLC/ESI-MS) and flow injection analysis—electrospray ionization—ion trap tandem mass spectrometry (FIA-ESI-IT-MS^n^) was developed to rapidly identify the proanthocyanidins present in the roots and barks of *P. biglobosa.*

Reactive oxygen species (ROS) and reactive nitrogen species (RNS), including free radicals such as superoxide radical anion, hydroxyl radicals, singlet oxygen, hydrogen peroxide and nitric oxide are continuously produced in human cells. Natural products have been attracting scientific interest due to their antioxidant and chemopreventive properties [33,34], as it is well-known that one of the main characteristics responsible for the antioxidant activity of a plant extract is its ability to scavenge free radicals, which can play a part in the protection against the harmful action of ROS. Thus, the aim of the present study was to characterize the proanthocyanidins profile as well as to evaluate the antiradical activity of the root and bark extracts of *P. biglobosa.*

## 2. Results and Discussion

### 2.1. FIA-ESI-IT-MS^n^ Analysis

Direct flow injection – electrospray ionization – mass spectrometry analysis can be used to establish the polyphenol profile of complex extracts [35], so technique was employed to obtain a preliminary qualitative metabolic fingerprint of the bark and root extracts of *P. biglobosa*, after a clean-up using LLE and SPE. The collected eluates were analyzed by ESI-IT-MS^n^ both in positive and negative ionization mode. The acidic nature of all the compounds present in extract of *P. biglobosa* makes negative ionization a good choice for obtaining high sensitivity [36]. Both extracts gave similar results (data not shown). Additional structural information was obtained by CID-MS-MS experiments. For identification and characterization of PAs, four main fragmentation mechanisms were observed: the retro-Diels-Alder (RDA), quinone methide (QM), heterocyclic ring fission (HFR) and the benzofuran-forming (BFF) mechanisms [22,37,38].

Some detected ions (**1**–**4**) have been previously reported and their fragmentations are summarized in Table 1. MS^2^ experiments with the precursor ions at *m/z* 481, 617 and 633 led to product ions at *m/z* 305 [M−H−176]^−^, 289 [M−H−152−176]^−^ and 305 [M−H−152−176]^−^, which suggested the presence of (*epi*)gallocatechin-*O*-glucuronide (**5**) [39], (*epi*)catechin-*O*-gallate-*O*-glucuronide (**6**) [40] and (*epi*)gallocatechin-*O*-gallate-*O*-glucuronide (**7**), respectively.

Precursor ions at *m/z* 577, 593 and 609, corresponding to catechin dimers, were attributed to (*epi*)catechin-(*epi*)catechin (**8**–**11**) [19], (*epi*)catechin-(*epi*)gallocatechin (**12**–**14**) and (*epi*)gallocatechin-(*epi*)gallocatechin (**15**–**16**) [37], respectively.

**Figure 1:**
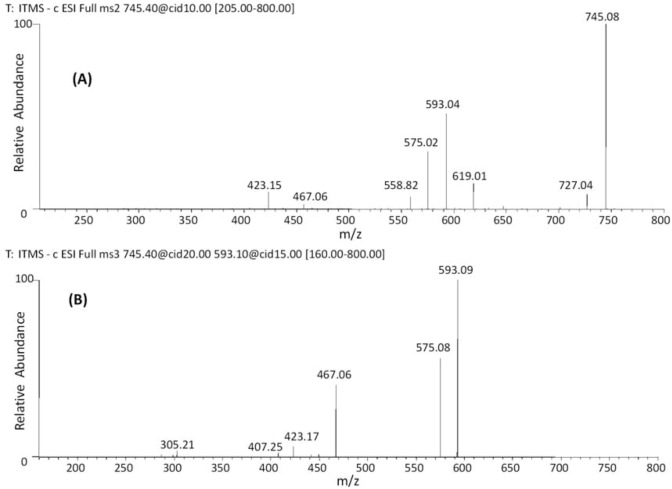
Negative ion ESI-MS^n^ spectra of B-type dimer. (**A**) MS^2^ of *m/z* 745 [M−H]^−^ and (**B**) MS^3^ of *m/z* 593 [M−H]^−^.

Catechin dimers esterified by galloyl units were identified at *m/z* 729 (**17**–**18**), 745 (**19**–**22**) and 761 (**23**–**26**) [37,41]. Second order fragmentation of the precursor ion [M−H]^−^ at *m/z* 745 (Figure 1A) produced fragments at *m/z* 619 [M−H−126]^−^, from an HRF fragment. Loss of 126 Da indicates that the A ring of the upper unit has a 1,3,5-trihydroxybenzene structure [22] and the fragment at *m/z* 593 [M−H−152]^−^ was assigned to the loss of one galloyl group. MS^3^ of the ion at *m/z* 593 (Figure 1B) produced fragments ions at *m/z* 575, 467 and 407, characteristic of an (*epi*)gallocatechin-(*epi*)catechin core with (*epi*)gallocatechin as the upper unit and esterified by the gallic acid in the lower unit [37,41].

**Table 1:** Peak number, retention time, *m/z* [M−H]^−^ ion, MS^n^ fragments of the compounds obtained by FIA-ESI-IT-MS^n^ and HPLC/ESI-IT-MS analysis of *P. biglobosa* roots and barks.

Peak No.	t_R_ (min)	[M−H]^−^, *m/z*	MS^2^	MS^3^	Proposed Names
Catechins monomers, esterified-catechin-*O*-gallate and *O*-glucuronide monomers (*)					
**1**	42.42	289	137 [M−H−152]^−^		(*epi*)catechin
**2**	24.98	305	179 [M−H−126]^−^		(*epi*)gallocatechin
**3**	43.33		167 [M−H−138]^−^		
			137 [M−H−168]^−^		
**4**	55.12	457	331 [M−H−126]^−^		(epi)gallocatechin-*O*-gallate
			305 [M−H−152]^−^		
			169 [M−H−288]^−^		
**5**	55.77	481	463 [M−H−18]^−^		(*epi*)gallocatechin-*O*-glucuronide
			305 [M−H−176]^−^		
			287 [M−H−176−18]^−^		
			313 [M−H−168]^−^		
**6**	55.74	617	599 [M−H−18]^−^	289 [M−H−152−176]^−^	(*epi*)catechin-*O*-gallate-*O*-glucuronide
			465 [M−H−152]^−^	271 [M−H−152−176−18]^−^	
			463 [M–H−170]^−^		
**7**	55.40	633	507 [M−H−126]^−^	463 [M−H−152−18]^−^	(*epi*)gallocatechin-*O*-gallate-*O*-glucuronide
			481 [M−H−152]^−^	305 [M−H−152−176]^−^	
				287 [M−H−152−176−18]^−^	
Catechins dimers (*)					
**8**	36.68	577	559 [M−H−18]^−^		(*epi*)catechin-(*epi*)catechin
**9**	46.27		451 [M−H−126]^−^		
**10**	48.40		425 [M−H−152]^−^		
**11**	49.89		407 [M−H−152−18]^−^		
			289 [M−H−288]^−^		
**12**	33.21	593	467 [M−H−126]^−^		(*epi*)catechin-(*epi*)gallocatechin
**13**	36.67		441 [M−H−152]^−^		
**14**	39.55		289 [M−H−304]^−^		
**15**	13.57	609	483 [M−H−126]^−^		(*epi*)gallocatechin-(*epi*)gallocatechin
**16**	22.46		441 [M−H−168]^−^		
			305 [M−H−304]^−^		
			303 [M−H−306]^−^		
Esterified catechin-*O*-gallate and *O*-glucuronide dimers – Group I (*)					
**17**	50.55	729	577 [M−H−152]^−^		(*epi*)catechin-(*epi*)catechin-*O*-gallate
**18**	54.93		559 [M−H−170]^−^		
			289 [M−H−440]^−^		
			287 [M−H−442]^−^		
**19**	38.09	745	619 [M−H−126]^−^	467 [M–H−152−126]^−^	(*epi*)gallocatechin-(*epi*)catechin-*O*-gallate
**20**	42.68		593 [M−H−152]^−^	407 [M–H−152−168−18]^−^	
**21**	47.44			575 [M−H−152−18]^−^	
**22**	49.89				
**23**	29.43	761	635 [M–H−126]^−^	591 [M−H−152−18]^−^	(*epi*)gallocatechin-(*epi*)gallocatechin-*O*-gallate
**24**	31.65		609 [M–H−152]^−^	483 [M−H−152−126]^−^	
**25**	36.87		593 [M–H−168]^−^	305 [M−H−152−304]^−^	
**26**	38.13				
**27**	45.18	785	659 [M−H−126]^−^	479 [M−H−168−138]^−^	(*epi*)gallocatechin-(*epi*)gallocatechin-*O*-glucuronide
**28**	47.48		617 [M−H−168]^−^	423 [M−H−168−176−18]^−^	
			591 [M−H−176−18]^−^	599 [M−H−168−18]^−^	
			481 [M−H−304]^−^		
Esterified catechin-*O*-gallate and *O*-glucuronide dimers – Group II (*)					
**29**	41.69	881	729 [M−H−152]^−^		(*epi*)catechin-*O*-gallate-(*epi*)catechin-*O*-gallate
**30**	54.71		577 [M−H−304]^−^		or
**31**	55.38				(*epi*)catechin-*O*-gallate–(*epi*)gallocatechin
**32**	54.93	897	745 [M−H−152]^−^		(*epi*)catechin-*O*-gallate-(*epi*)gallocatechin-*O*-gallate
**33**	55.28		727 [M−H−170]^−^		
**34**	49.44	913	761 [M–H−152]^−^		(*epi*)gallocatechin-*O*-gallate-(*epi*)gallocatechin-*O*-gallate
**35**	50.62		787 [M−H−126]^−^		
			743 [M−H−170]^−^		
**36**	54.88	937	919 [M−H−18]^−^	641 [M−H−170−126]^−^	(*epi*)gallocatechin-*O*-gallate-(*epi*)gallocatechin-*O*-glucuronide
**37**	55.27		811 [M−H−126]^−^	629 [M−H−170−138]^−^	
			785 [M−H−152]^−^	591 [M−H−152−176−18]^−^	
			767 [M−H−170]^−^	599 [M−H−176−168]^−^	
Catechin trimer (*)					
**38**	55.54	865	739 [M−H−126]^−^		(*epi*)catechin-(*epi*)catechin-(*epi*)catechin
			713 [M−H−152]^−^		
			695 [M−H−152−18]^−^		
			577 [M−H−288]^−^		
			425 [M−H−288−152]^−^		
**Esterified catechin-*O*-gallate and *O*-glucuronide trimers (#)**					
**39**	54.95	1017	n.a.	n.a.	(*epi*)catechin-(*epi*)catechin-(*epi*)catechin-*O*-gallate
**40**	40.75	1049	n.a.	n.a.	(*epi*)gallocatechin-(*epi*)gallocatechin-(*epi*)gallocatechin-*O*-gallate
**41**	45.73				
**42**	54.86				
**43**	55.22				
**44**	46.55	1089	n.a.	n.a.	(*epi*)gallocatechin-(*epi*)gallocatechin-(*epi*)gallocatechin-*O*-glucuronide
**45**	55.52				
**Catechin tetramers (#)**					
**46**	55.25	1153	n.a.	n.a.	(*epi*)catechin-(*epi*)catechin-(*epi*)catechin-(*epi*)catechin
**47**	55.23	1169	n.a.	n.a.	(*epi*)catechin-(*epi*)catechin-(*epi*)catechin-(*epi*)gallocatechin
**48**	55.32	1185	n.a.	n.a.	(*epi*)catechin-(*epi*)catechin-(*epi*)gallocatechin-(*epi*)gallocatechin
**49**	54.83	1201	n.a.	n.a.	(*epi*)catechin-(*epi*)gallocatechin-(*epi*)gallocatechin-(*epi*)gallocatechin
**50**	54.90	1217	n.a.	n.a.	(*epi*)gallocatechin-(*epi*)gallocatechin-(*epi*)gallocatechin-(*epi*)gallocatechin

(*) ions obtained by FIA-ESI-IT-MS^n^. (**#**) ions obtained by HPLC-ESI-IT-MS. n.a. not analyzed.

**Figure 2:**
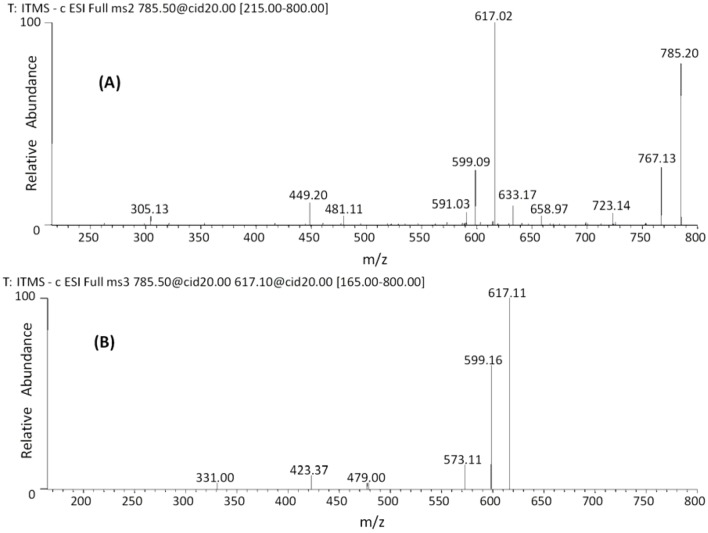
Negative ion ESI-MS^n^ spectra of B-type dimer. (**A**) MS^2^ of *m/z* 785 [M−H]^−^ and (**B**) MS^3^ of *m/z* 617 [M−H]^−^.

**Figure 3:**
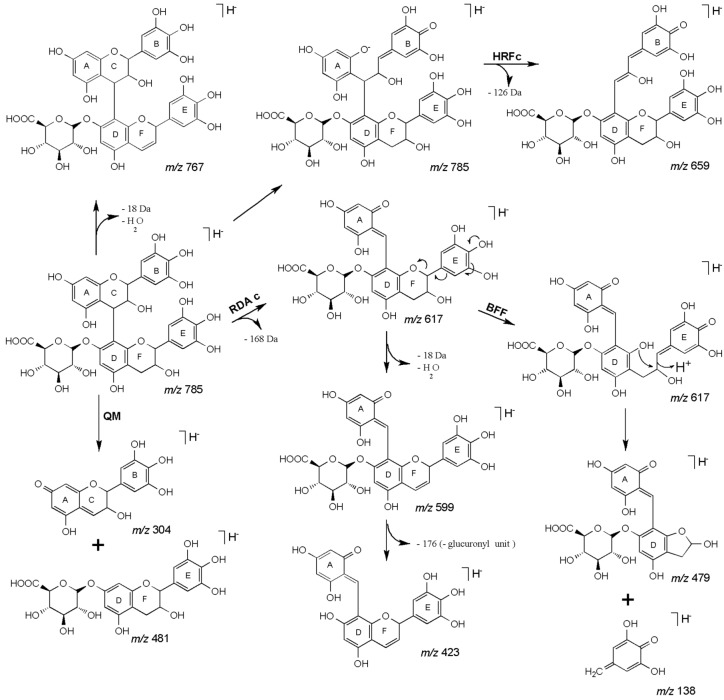
Characteristic fragmentation pathways of B-type proanthocyanidins proposed for the glucuronide dimer identified in the enriched fractions from roots of *P. biglobosa*.

The MS spectrum of the *m/z* 785 (Figure 2A) showed the base peak at *m/z* 617 [M−H−168]^−^ from an RDA fragment, neutral loss of 168 Da indicates that the B ring of the upper unit has a pyrogalloyl group [38]. The ion at *m/z* 659 [M−H−126]^−^ is due to the loss of an HRF fragment; the ion at *m/z* 591 [M−H−194]^−^ is assigned to the loss of a glucuronide moiety (176 Da) [39] followed by the loss of a water molecule (18 Da) and the one at *m/z* 481 [M−H−304]^−^, assigned to the loss of one (*epi*)gallocatechin unit through a QM fission. The MS^3^ spectrum (Figure 2B) of the ion at *m/z* 617 produced ions at *m/z* 599 [M−H−168−18]^−^, loss of an RDA fragment plus water as major ion. The diagnostic fragment ion at *m/z* 479 [M−H−168−138]^−^ could result from the loss of an RDA fragment (168 Da) plus the loss of an BFF fragment (138 Da) [38] from the lower unit of the *m/z* 617 ion. The important ion at *m/z* 423 [M−H−168−194]^−^ was assigned to a sequential loss of an RDA fragment (168 Da), a water molecule (18 Da) and a loss of a glucuronide moiety (176 Da). These data confirm the (*epi*)gallocatechin-*O*-glucuronide as the lower unit and the (*epi*)gallocatechin as the upper unit. Finally, the molecule was characterized as an *O*-glucuronide of (*epi*)gallocatechin-(*epi*)gallocatechin (**27**–**28**) and this is the first report about this species. Figure 3 shows the main fragmentation pathways.

Another series are represented by the signals at *m/z* 881, 897, 913 and 937 [M−H]^−^ which indicate that the corresponding compounds are mixed dimers consisting of (*epi*)gallocatechin and/or (*epi*)catechin units, with galloyl residues to three first *m/z* and galloyl and glucuronide residues to *m/z* 937.

Fragmentation of the precursor ion at *m/z* 881 produced ions at *m/z* 729 [M−H−152]^−^ corresponding to the loss of one gallate moiety and at *m/z* 577 [M−H−304]^−^, that can corresponds to the loss of two gallate moieties or a gallocatechin residue. Thus, we suggested that this compound could be a dimer constituted of two (*epi*)catechin-*O*-gallate units [41] or (*epi*)catechin-*O*-gallate–(*epi*)gallocatechin (**29**–**31**).

The MS^2^ fragmentation of the precursor ion at *m/z* 897 showed fragment ions at *m/z* 745 [M−H−152]^−^, assigned to the loss of a galloyl group, at *m/z* 727 [M−H−170]^−^, due to the loss of a gallic acid molecule. The products ions at *m/z* 287 and 305 Da observed in the MS^2^ spectra after a QM fission suggest that the (*epi*)catechin core is the upper unit [37], identifying the compounds **32**–**33**.

Fragmentation of the precursor ion at *m/z* 913 produced fragment ions at *m/z* 761 [M−H−152]^−^, due to the loss of one galloyl group, and at *m/z* 743 [M−H−170]^−^, due to the loss of a gallic acid molecule. The ion observed at *m/z* 787 [M−H−126]^−^ can result to the loss of an HRF fragment. Thus, this metabolite was tentatively identified as a dimer constituted of (*epi*)gallocatechin-*O*-gallate moieties (**34**–**35**) [41].

**Figure 4:**
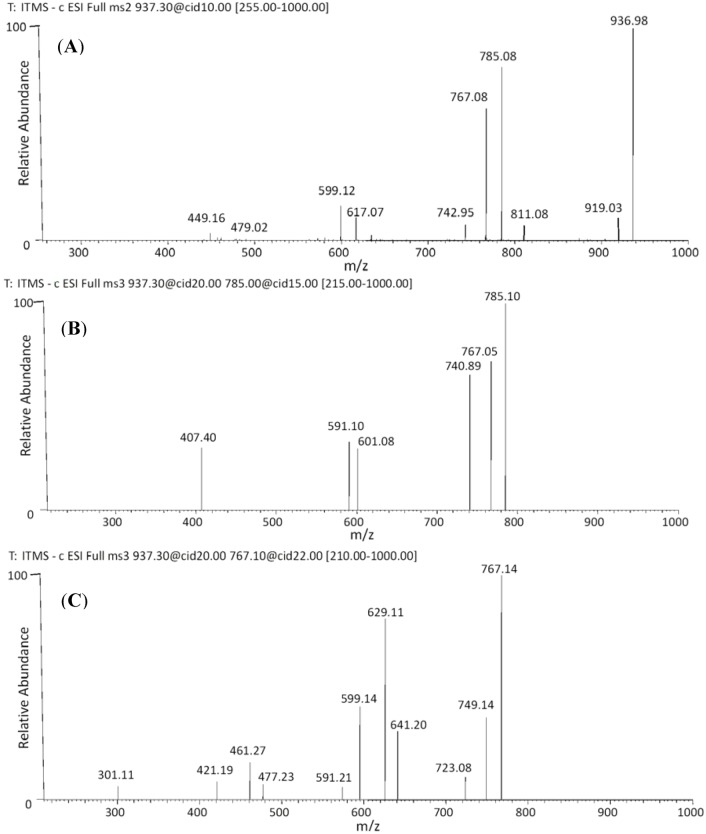
Negative ion ESI-MS^n^ spectra of B-type dimer. (**A**) MS^2^ of *m/z* 937 [M−H]^−^, (**B**) MS^3^ of *m/z* 785 [M−H]^−^ and (**C**) MS^3^ of *m/z* 767 [M−H]^−^.

The MS^2^ spectra of *m/z* 937 (Figure 4A) shows the main product ions at *m/z* 785 [M−H−152]^−^ assigned to the loss of a galloyl group and at *m/z* 767 [M−H−170]^−^ assigned to the loss a gallic acid. The MS^3^ spectra (Figure 4B) of the *m/z* 785 produced ion at *m/z* 591 [M−H−152−194]^−^ assigned to the loss of a galloyl group (152 Da) plus the loss of a glucuronide moiety (176 Da) followed by the loss of a water molecule (18 Da). The MS^3^ spectra (Figure 4C) of the *m/z* 767 produced fragment ions at *m/z* 641 [M−H−170−126]^−^ due to the loss of a gallic acid (170 Da) plus the loss of an HRF fragment (126 Da); at *m/z* 629 [M−H−170−138]^−^ which could result from the loss of a gallic acid (170 Da), plus a BFF at the lower unit of the *m/z* 767 ion. Hence this dimer was characterize as an (*epi*)gallocatechin-*O*-gallate-(*epi*)gallocatechin-*O*-glucuronide (**36**–**37**) and it is reported for the first time.

The trimeric proanthocyanidin (**38**) (*m/z* 865 [M−H]^−^) were analyzed in the same way. First investigations by ESI in the negative ion mode showed that the fragmentation was similar to that observed to the dimers. The MS^2^ shows a sequence of the fragmentation characteristics of these compounds: at *m/z* 739 [M−H−126]^−^ from the loss of an HRF fragment (126 Da), at *m/z* 713 [M−H−152]^−^ from the loss of an RDA fragment, the neutral loss of 152 Da indicates that the B ring of the upper unit has a catechol group [38] at *m/z* 577 [M−H−288]^−^, was assigned to interflavanic bond cleavage and at *m/z* 451 [M−H−288−126]^−^, which could be formed after the interflavanic fission plus the loss of an HRF fragment (126 Da). Also, similar to the behavior in the positive ion mode described [42] a prominent ion was observed at *m/z* 575 [M−H−290]^−^, assigned to interflavanic bond cleavage following the QM mechanism. This precursor ion was then identified as a trimer of (*epi*)catechin.

### 2.2. HPLC/ESI-IT-MS Analysis

**Figure 5:**
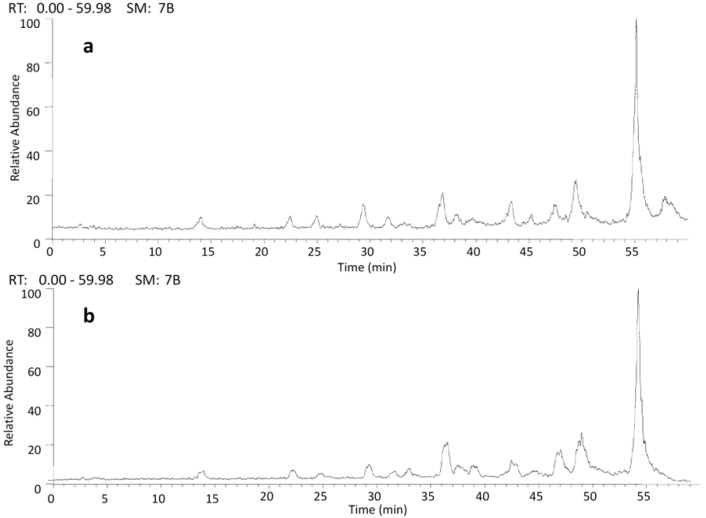
Negative ion HPLC/ESI-IT-MS analysis of proanthocyanidins present in *P. biglobosa.* (**a**) total ion chromatogram of roots and (**b**) total ion chromatogram of barks.

It has been shown that catechins, which are monomeric units in PAs, are found in plant extracts and almost always identified as catechin and/or (*epi*)catechin, probably due to stereoselectivity of the enzymes involved in the biosynthesis of these substances [43]. An analytical strategy based on the online HPLC/ESI-IT-MS approach was applied to investigate the presence of isomers of catechins derivatives in *P. biglobosa* extracts since FIA-ESI-IT-MS^n^ cannot distinguish between stereoisomers [37]. The results obtained revealed a different number of PAs isomers in these extracts and the presence of other B-type PAs which have also been detected in the roots and barks of the species (Figure 5). The UV spectra of all peaks exhibited absorption maxima bands at 205–215 nm and 270–285 nm, typical of proanthocyanidins [19], confirming the preliminary phytochemical study in both extracts.

Deprotonated molecules corresponding to proanthocyanidins monomers were detected at t_R_ = 42.42 min [*m/z* 289 (**1**)], at t_R_ = 55.12 min [*m/z* 457 (**4**)], at t_R_ = 55.77 min [*m/z* 481 (**5**)], at t_R_ = 55.74 min [*m/z* 617 (**6**)], and at t_R_ = 55.40 min [*m/z* 633 (**7**)]. Noteworthy, two isomers at *m/z* 305 (**2**–**3**) were identified in the extracted ion chromatogram of roots and barks at the retention time of 24.98 and 43.33 min.

Compounds isomeric with catechin dimers were detected for precursor ions at *m/z* 577 (**8**–**11**), 593 (**12**–**14**) and 609 (**15**–**16**). Another series, with isomeric forms, were identified as esterified catechin-gallate and glucuronide dimers for ions at *m/z* 729 (**17**–**18**), 745 (**19**–**22**), 761 (**23**–**26**), 785 (**27**–**28**), 881 (**29**–**31**), 897 (**32**–**33**), 913 (**34**–**35**) and 937 (**36**–**37**). The retention time of these isomeric compounds were listed in Table 1.

The trimeric proanthocyanidin (**38**) at *m/z* 865 [M−H]^−^ was confirmed at t_R_ = 55.54 min.

Using the same analytical approach, it was possible to detect from the total ion current chromatogram (TIC) *m/z* values corresponding to gallate and glucuronide trimer with their isomers. The precursor ions at *m/z* 1,017 (**39**) (t_R_ = 54.95 min) and *m/z* 1,049 (**40**–**43**) [(t_R_ = 40.75; 45.73; 54.86 and 55.22 min), four isomers] were assigned to trimers of (*epi*)catechin and (*epi*)gallocatechin esterified with a gallic acid unit respectively, as postulated by Lazarus [44]. On the other hand, the precursor ion at *m/z* 1,089 (**44**–**45**) (t_R_ = 46.55 and 55.52 min, two isomers) was tentatively assigned to a glucuronide trimer namely a trimer of (*epi*)gallocatechin-*O*-glucuronide present in the extract with its stereoisomer. The *m/z* values corresponding to tetramer molecular weights at *m/z* 1,153 (**46**) (t_R_ = 55.25 min), *m/z* 1,169 (**47**) (t_R_ = 55.23 min), *m/z* 1,185 (**48**) (t_R_ = 55.32 min), *m/z* 1,201 (**49**) (t_R_ = 54.83 min) and *m/z* 1,217 (**50**) (t_R_ = 54.90 min) were assigned to tetramers containing from zero to four trihydroxylated units, respectively [36].

Finally, tetramers were the highest *m/z* values that could be detected by electrospray in these fractions of roots and barks of *P. biglobosa*. Thus, proanthocyanidins present in this plant are constituted, of procyanidins, with mixed procyanidin-prodelphinidin structures and (*epi*)gallocatechin oligomers. In order to identify higher polymers, these were concentrated in the aqueous fraction [45] (as described in subsection 3.3 of the Experimental), one analytical alternative for the characterization of polymers includes the matrix-assisted laser desorption ionization/time of flight (MALDI-TOF) mass spectrometry, that permits the identification of tannins with higher degree of polymerization [46].

The antiradical activity of the CH_2_Cl_2_/MeOH (1:1, v/v) extracts and the ethyl acetate and aqueous fractions of the barks and roots was evaluated using a DPPH assay, with gallic acid and quercetin as antiradical reference compounds.

The results showed that all the extracts and fractions of roots and barks exhibited good free radical scavenging activity compared to the references quercetin (IC_50_ 20.6 ± 0.1 μg·mL^−1^) and gallic acid (IC_50_ 16.2 ± 0.1 μg·mL^−1^). The aqueous fraction of the roots was the one that had the highest free radical sequestration capacity (IC_50_ 31.5 ± 0.1 μg·mL^−1^), followed by the aqueous fraction of the barks (IC_50_ = 37.1 ± 0.1 μg·mL^−1^), the EtOAc fractions of the barks (IC_50_ = 37.8 ± 0.3 μg·mL^−1^), the CH_2_Cl_2_/MeOH (1:1, v/v) extract of the roots (IC_50_ = 41.7 ± 0.1 μg·mL^−1^), the CH_2_Cl_2_/MeOH (1:1, v/v) extract of the barks (IC_50_ = 44.6 ± 0.1 μg·mL^−1^) and finally the EtOAc fraction of the roots (IC_50_ = 53.1 ± 0.1 μg·mL^−1^). The aqueous fraction showed more activity due the presence of oligomeric proanthocyanidins.

The remarkable antioxidant activities exhibited by all the *P. biglobosa* extracts and fractions tested could be associated with the presence of (*epi*)catechin derivatives, particularly (*epi*)gallocatechin-*O*-gallate [47] and condensed tannins, well known as being very powerful antioxidants [25]. Other polyphenols, such as flavonoids and phenolic acids, are strong antioxidants too. These chemical classes are responsible for the high antiradical activity of extracts obtained from brewery waste streams [48].

## 3. Experimental

### 3.1. General

HPLC-grade methanol and acetic acid were purchased from J.T. Baker (Baker Mallinckrodt, Phillipsburg, NJ, USA). CH_2_Cl_2_ and EtOAc were purchased from Tedia (Rio de Janeiro, RJ, Brazil). HPLC-grade water was prepared using a Millipore (Bedford, MA, USA) Milli-Q purification system. The RP18 cartridge is a Phenomenex Strata C18-E, 55 µm, 70 Å, 500 mg·3 mL^−1^. The filter membrane (0.45 µm) was of nylon.

### 3.2. Plant Material

Barks and roots of *P. biglobosa* were collected in October 2008 at Ngaoundere-Cameroon, Africa, and authenticated *in loco* by Professor Dr. Pierre Marie Mapongmetsem and confirmed in the National Herbarium through the voucher sample (n° 58980 HNC) collected by Dr. Francois Villiers from National Museum of Natural History of Paris. 

### 3.3. Extraction and Sample Preparation

Dried and powdered barks (1 g) and roots (1 g) of *P. biglobosa* were macerated separately for a week, at room temperature, with 100 mL of a 1:1 (v/v) mixture of CH_2_Cl_2_/MeOH. The solutions were evaporated to dryness under vacuum to give 100 mg (10%) of crude bark and root extracts. In order to minimize the interference of very high order polymeric compounds, a combination of liquid-liquid extraction (LLE) and solid-phase extraction (SPE) was employed [45]. In the first step, 100 mg of each extract were, separately, submitted to LLE between EtOAc and H_2_O (50 mL of each solvent). After drying, the EtOAc fractions afforded 30 mg of each sample. An aliquot (10 mg) of the EtOAc fraction of each extract were submitted to the SPE using RP18 cartridge, eluted with H_2_O/MeOH 8:2 (v/v) (5 mL). The eluate was filtered through the nylon membrane and directly analyzed by ESI-IT-MS^n^ as well as by HPLC/ESI-MS. 

### 3.4. Mass Spectrometric Analysis

#### 3.4.1. FIA-ESI-IT-MS^n^

Flow injection analysis (FIA) was performed using a ThermoFisher Scientific ion trap mass spectrometer (San Jose, CA, USA) equipped with an electrospray ionization source. The MS and MS/MS analysis in negative ion mode were selected after calibration infusing a standard solution of (+)-catechin (1 µg·mL^−1^ in methanol) at a flow rate of 5 µL·min^−1^ and working under the following conditions: capillary voltage −31 V, spray voltage 5 kV, tube lens offset 75 V, capillary temperature 300 °C, sheath gas (N_2_) flow rate 8 (arbitrary units). Negative ion mass spectra were recorded in the range *m/z* 100–2000 Da. The first event was a full - scan mass spectrum to acquire data on ions in the *m/z* range. The second scan event was an MS/MS experiment performed by using data-dependent scan that was carried out on deprotonated molecules from the compounds at collision energy of 30% and activation time of 30 ms. Data acquisition and processing were performed using the Xcalibur software.

#### 3.4.2. HPLC/ESI-IT-MS

Analysis were performed by HPLC/ESI-MS using a SURVEYOR MS micro HPLC system coupled on-line with an LCQ Fleet ion trap mass spectrometer (Thermo Fisher Scientific). HPLC separation was conducted on a Synergi Hydro RP-18 column (250 × 4.6 mm, L × i.d.; 4 µm, Phenomenex), at a flow rate of 800 µL·min^−1^. Gradient elution was performed by using H_2_O (A) and MeOH (B), both added of 0.1% acetic acid, as mobile phases. After a 5 min hold at 5% B, elution was performed according to the following conditions: from 5% B to 25% B in 50 min; 25% to 100% B in 60 min. The column effluent was split into two by means of a “T junction” placed after the chromatographic column and analyzed “on-line” both by ESI/MS and UV-DAD; 80% of the effluent sent to the UV-DAD detector; 20% of the effluent was analyzed by ESI/MS in negative ion mode. The mass spectra were acquired and processed using the Xcalibur software (version 1.3) provided by the manufacturer. Capillary voltage 20 V, capillary temperature 275 °C, nitrogen as drying gas at a flow rate 4 (arbitrary units) and as nebulizing gas, ion spray voltage 5 kV, tube lens offset 80 V, sweep gas at a flow rate 15 (arbitrary units). Data were acquired in MS^1^ scanning modes in the range of *m/z* 100–2,000 Da. The ESI interface and MS parameters were optimized to obtain maximum sensitivity. PAs were identified based on observation of the *m/z* values of their deprotonated molecules.

### 3.5. Antiradical Activity Determination

DPPH assays were used to measure the free radical scavenging potential of the extracts [49]. The CH_2_Cl_2_/MeOH (1:1, v/v) extracts and ethyl acetate and aqueous fractions of the barks and roots, separately, were obtained by partition using 2.5 mg of each in 10 mL of the H_2_O/MeOH (2:8, v/v) and them dilutions were made until the concentrations of 6.25, 12.5, 25, 50, 100 and 200 mg·mL^−1^. Solutions of 0.004% of DPPH in H_2_O/MeOH (2:8, v/v) were used, which were mixed with the sample solution being examined. Aliquots (20 μL) of the sample were added to the DPPH solutions (200 μL). After 30 min of reaction, absorbance was immediately recorded at 517 nm. Quercetin and gallic acid (Sigma Aldrich^®^, St Louis, MO, USA) standard solutions were prepared and analyzed under the same conditions. The results were expressed as 50% inhibitory concentration (IC_50_ in μg·mL^−1^). All analyses were the mean of triplicate measurements ± standard deviation. Lower IC_50_ value indicates higher antioxidant activity.

## 4. Conclusions

To the best of our knowledge no study had previously described the tannins profile of *Parkia biglobosa.* Thereby, with the aim of providing further scientific contributions to support and improve African economy, we decided to carry out an analytical study to define the metabolic fingerprinting of this plant.

Effective, rapid and sensitive HPLC/ESI-IT-MS and FIA-ESI-IT-MS^n^ methods were developed for characterizing the oligomeric constituents in the plants. The work established the profile of proanthocyanidins in the extract of roots and barks of *P. biglobosa*. Mass spectral data indicated that B-type oligomers are present, namely procyanidins and prodelphinidins, with their gallate and glucuronide derivatives, some of them in different isomeric form. With this method, some proanthocyanidins with novel structures were determined for the first time. Tandem Mass Spectrometry (MS^n^) was demonstrated to be a useful analytical tool of choice for characterizing PAs, since more information about the structural details of the different molecules can be elucidated from the fragmentation of the precursor ions. The occurrence of proanthocyanidins is in accordance with chemical constituents of this genus, since tannins were previously identified in *P. clappertoniana* [14].

The antiradical activity of the extracts and the fractions was evaluated using the DPPH assay, with gallic acid and quercetin as antiradical reference compounds. The study suggests that the high antiradical activity of extracts and fractions from *P. biglobosa* can be attributed to the proanthocyanidin derivatives present in this plant.
